# Microcrystalline array structures induced by heat treatment of friction-transferred organic semiconductor films

**DOI:** 10.1038/s41598-019-46212-w

**Published:** 2019-07-05

**Authors:** Yuhi Inada, Masashi Koda, Yuji Urabe, Toshifumi Katagiri, Takeshi Yamao, Yuji Yoshida, Shu Hotta

**Affiliations:** 1 Faculty of Materials Science and Engineering, Kyoto Institute of Technology, Kyoto, 606-8585 Japan; 2 Sumitomo Seika Chemicals Co., Ltd., Harima, Hyogo, 675-0145 Japan; 3 National Institute of Advanced Industrial Science and Technology (AIST), Tsukuba, 305-8565 Japan

**Keywords:** Electronic and spintronic devices, Electronic materials, Optical materials, Electronic properties and materials

## Abstract

The correlation between molecular orientation and optoelectrical properties is most critical to the future design of molecular materials. We made highly-anisotropic microcrystalline array structures with an organic semiconductor, a methoxy-substituted thiophene/phenylene co-oligomer (TPCO), by depositing it on friction-transferred poly(tetrafluoroethylene) (PTFE) layers fabricated on substrates with several heat treatments. Polarising microscope observation, polarised emission and absorption spectra measurements indicated that the TPCO molecules aligned along the drawing direction of PTFE. Using these films, we fabricated two types of field-effect transistors (FETs) and compared them with those using non-heated TPCO films which provide aligned pleats structures. Ones had the channel length direction parallel to the drawing direction of PTFE and the others had the channel length direction perpendicular to that drawing direction. As for the microcrystalline array films, the mobility ratio of the former FET to that of the latter device was about 27 in the saturation region, while the emission polarisation ratio was 4.5. The heat treatment promoted the crystal growth to enhance the mobility while retaining the high anisotropy. The results demonstrate that the heat treatments of the TPCO films on the friction-transferred layers were useful for controlling crystallinity and orientation of the molecules.

## Introduction

Organic materials are expected to be applied to unique devices including electronic papers and flexible displays by virtue of useful characteristics e.g. light-weight, flexible and printable. In applying the organic materials to devices, electronic and optical properties are affected by not only the characteristics of these materials but also structural anisotropy and spatial overlap of the molecules such as crystallinity and orientation^1^. These are also influenced by the quality and morphology of the organic films and the device structures^2^.

For molecular orientation, we can use the friction-transfer technique^3^. This readily produces oriented polymer films by drawing a polymer block on a substrate without using solvents and vacuum^1,4–7^. Since Wittmann and Smith reported friction-transferred poly(tetrafluoroethylene) (PTFE) films in 1991^3^, this technique was developed and widely used as the effective molecular orientation method^8–11^. In the cases of organic light-emitting diodes, the device performance was improved by controlling molecular orientation for carriers to transport effectively perpendicular to the substrate^7^. As for organic field-effect transistors (OFETs), the molecular orientation for the effective lateral carrier transport was preferred^1^.

Previously we made highly oriented films of thiophene/phenylene co-oligomers (TPCOs) by depositing them on friction-transferred PTFE layers fabricated on substrates^12^. Even though these TPCO films indicated the anisotropic optical and electronic properties^12^, it is desired to enhance the crystallinity as well.

In the present studies, we have fabricated highly-anisotropic microcrystalline array structures by applying heat treatment to orientation films of a methoxy-substituted TPCO deposited on friction-transferred PTFE layers to enhance the crystallinity and carrier mobility while retaining the high anisotropy.

We examined optical properties of the resulting films, carried out their microscopies and measured current-voltage (*I*–*V*) characteristics of OFETs made of these films. For comparison, we also prepared the OFETs with orientation films on the PTFE layers with or without heat treatment during TPCO deposition.

## Results and Discussion

### Sample preparation

**Figure 1:**
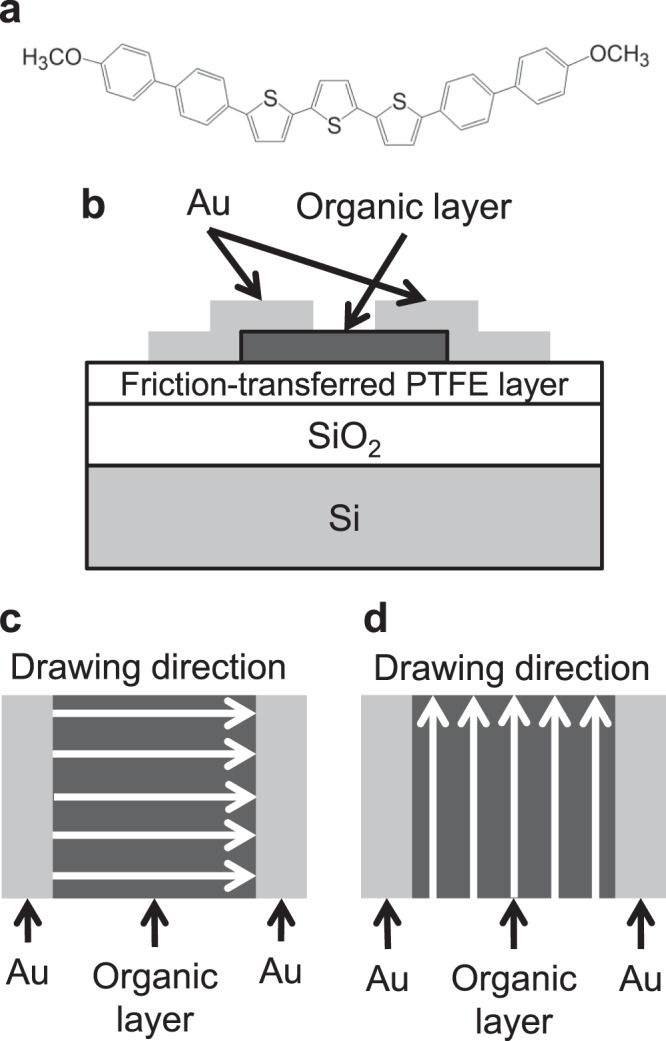
(**a**) Structural formula of BP3T-OMe. (**b**) Schematic structure of the OFET. Parts of top views indicating drawing directions of PTFE (**c**) parallel and (**d**) perpendicular to the channel length direction of the OFET.

We chose 5,5″-bis(4′-methoxybiphenyl-4-yl)-2,2′:5′,2″-terthiophene (BP3T-OMe), that is 5,5″-bis(4-biphenylyl)-2,2′:5′,2″-terthiophene (BP3T)^13^ substituted with methoxy groups at both molecular terminals. Figure 1(a) shows the structural formula of BP3T-OMe. The synthetic method is described in the Supplementary Information. We prepared friction-transferred PTFE layers on Si substrates covered with a SiO_2_ layer and quartz substrates. We deposited 100-nm-thick BP3T-OMe on the untreated SiO_2_/Si substrate, the friction-transferred SiO_2_/Si substrates and the friction-transferred quartz substrates. After film deposition, we heated untreated and friction-transferred PTFE substrates at 100, 150 or 200 °C for ~1 h. Hereafter, we call this treatment “postheat” treatment. As another heat treatment, we deposited in a vacuum a 100-nm-thick BP3T-OMe film on the friction-transferred PTFE substrates that were preliminarily heated and kept at 70 or 100 °C in a vacuum (substrate heating). Table 1 shows a summary of the film fabrication conditions. The table includes sample numbers.

**Table 1:** Preparation conditions of organic thin films.

Sample No.	Friction-transferred film	Postheat treatment (°C)	Substrate heating (°C)
1	No	—	—
2	No	100	—
3	No	150	—
4	No	200	—
5	Yes	—	—
6	Yes	100	—
7	Yes	150	—
8	Yes	200	—
9	Yes	—	70
10	Yes	—	100

Note that in Samples 1–4 the substrate was not friction-transferred with PTFE.

We fabricated OFETs using the deposited films on the friction-transferred SiO_2_/Si substrates [Fig. 1(b)]. We fabricated two types of devices where the drawing directions of PTFE were parallel and perpendicular to the channel length direction [see Fig. 1(c,d)]. Here, the channel length direction means the direction perpendicular to the parallel edges of the Au layers (used as source and drain contacts). We also fabricated an OFET with the BP3T-OMe film on the substrate without PTFE layer.

### Polarising microscope observation

**Figure 2:**
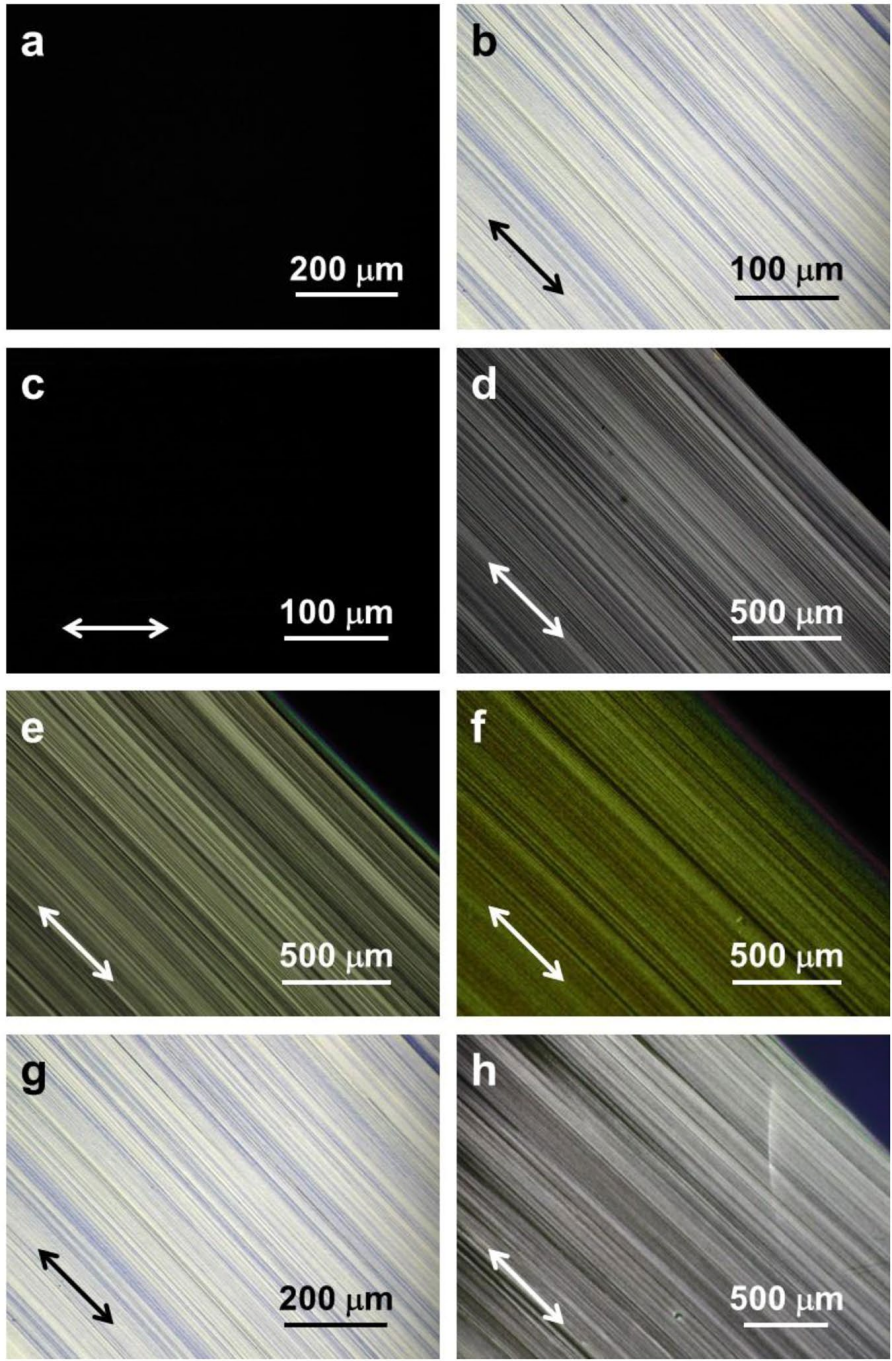
Polarising micrographs of the BP3T-OMe films. (**a**) Sample 1. (**b**) Diagonal and (**c**) distinction positions of Sample 5. Diagonal positions of (**d**) Sample 6, (**e**) Sample 7, (**f**) Sample 8, (**g**) Sample 9 and (**h**) Sample 10. The polarising directions of the analyser and polariser are parallel to vertical and horizontal directions, respectively. Double-headed arrows indicate the drawing direction.

The polarising micrographs of the BP3T-OMe film deposited on the substrates without a PTFE layer (Sample 1) were dark at any position under the crossed Nicols [Fig. 2(a)], indicating the isotropic film. Figure 2(b,c) show polarising micrographs of the BP3T-OMe film deposited on the friction-transferred PTFE layer without heat treatment (Sample 5). The bright lines of the film image [Fig. 2(b)] disappeared when the drawing direction accorded with the polarising direction of the polariser or the analyser [Fig. 2(c)], indicating the molecular orientation of the film.

The same molecular orientations were observed in the postheat-treated films at 100, 150 and 200 °C and in the films deposited on the heated substrates at 70 and 100 °C (Samples 6–10) [Fig. 2(d–h)]. The postheat-treated film images [Samples 7 and 8, Fig. 2(e,f)] were dark as well as the non-polarising micrographs.

### X-ray diffraction

X-ray diffraction (XRD) patterns of the BP3T-OMe films deposited under the various conditions are shown in Fig. S1 (Supplementary Information). We summarised major peak positions and plane separations in Table S1 (Supplementary Information).

For Sample 1 (without a friction-transferred layer or heat-treatment), we observed first-, third-, fourth-, fifth-, eighth- and ninth-order peaks related to a long plane separation of ~3.2 nm [marked with asterisks in Fig. S1(a), Supplementary Information] and first-order peaks associated with short separations of ~0.46 (a triangle) and ~0.38 nm (a diamond). If the crystal system of BP3T-OMe is assumed to be monoclinic or orthorhombic and the molecules stand against the crystal plane as shown in unsubstituted TPCOs or methoxy-substituted TPCOs^14–17^, ~3.2 nm is a half of the distance between the planes composed of the crystallographic short and intermediate axes. This distance agrees with the calculated molecular length of BP3T-OMe (3.6 nm). The short separations ~0.38 and ~0.46 nm correspond to the length of the crystallographic intermediate axis and the distance between the hypotenuse and the point of the right angle made by the crystallographic short and intermediate axes, respectively. The results indicated that the BP3T-OMe molecules both stood and lay down on the substrate in Sample 1.

As for Sample 5 (with a friction-transferred layer without heat-treatment), the weak first-order peak associated with the separation ~3.2 nm was observed at 2*θ* = 2.77°. The peak associated with ~0.38 nm (2*θ* = 23.60°) increased [see Fig. S1(b), Supplementary Information] compared with that in Sample 1. This showed that the proportion of the molecules lying down (standing) on the substrate increased (decreased) and that the lying-down molecules were dominant in Sample 5.

Although the same was observed in the postheat-treated films and the films deposited on the heated substrates [see Fig. S1(c,e–g), Supplementary Information], the peak associated with ~0.38 nm disappeared and the peak at 2*θ* = 28.05° became noticeable in Sample 7 [postheat-treated at 150 °C, Fig. S1(d), Supplementary Information]. This suggests that the molecular reorientation occurred at around 150 °C in the postheat-treated films.

In Sample 8, the diffraction peak associated with ~0.38 nm was dominant at 2*θ* = 23.54° [Fig. S1(e), Supplementary Information]. This indicates that the molecules were aligned so that the crystallographic intermediate axis stood against the substrate surface. This crystal alignment was clarified by scanning electron microscope (SEM) observation (vide infra).

### Polarised emission spectra

The polarised emission spectra are shown in Fig. S2 (Supplementary Information). For all films, the parallel emissions relative to the drawing direction were larger than the perpendicular ones. This suggests that the direction of transition dipole moment (the molecular long axis) of BP3T-OMe was in agreement with the drawing direction. We calculated their intensity ratios of the parallel component to the perpendicular one around the major peak positions and summarise them in Table S2 (Supplementary Information).

Sample 5 showed peaks at 573 and 615 nm [see Fig. S2(a), Supplementary Information] and the ratios were 2.7 and 2.5, respectively. For the postheat-treated films at 100 (Sample 6) and 150 °C (Sample 7) [Fig. S2(b,c), Supplementary Information) these ratios decreased to 1.7 and 2.4 at 590 and 592 nm, respectively. Sample 8 [postheat-treated at 200 °C, see Fig. S2(d), Supplementary Information] showed the ratio 4.5 at 591 nm. This value was ~1.7 times larger than that of Sample 5.

As for the films deposited on the heated substrates, Sample 9 [at 70 °C, see Fig. S2(e), Supplementary Information] showed the ratio of 5.0 at 572 nm and Sample 10 [at 100 °C, see Fig. S2(f), Supplementary Information] indicated 4.9 at 571 nm and a maximum of 5.2 at 614 nm. The value 5.2 was almost twice as large as that of Sample 5. This indicates that the BP3T-OMe molecular long axis was highly oriented in the drawing direction in Sample 10.

Among the samples, Samples 8–10 show the higher polarisation ratios ~5 (see Table S2, Supplementary Information). This indicates that the molecules were well-aligned in these films.

### Polarising absorption spectra

**Figure 3:**
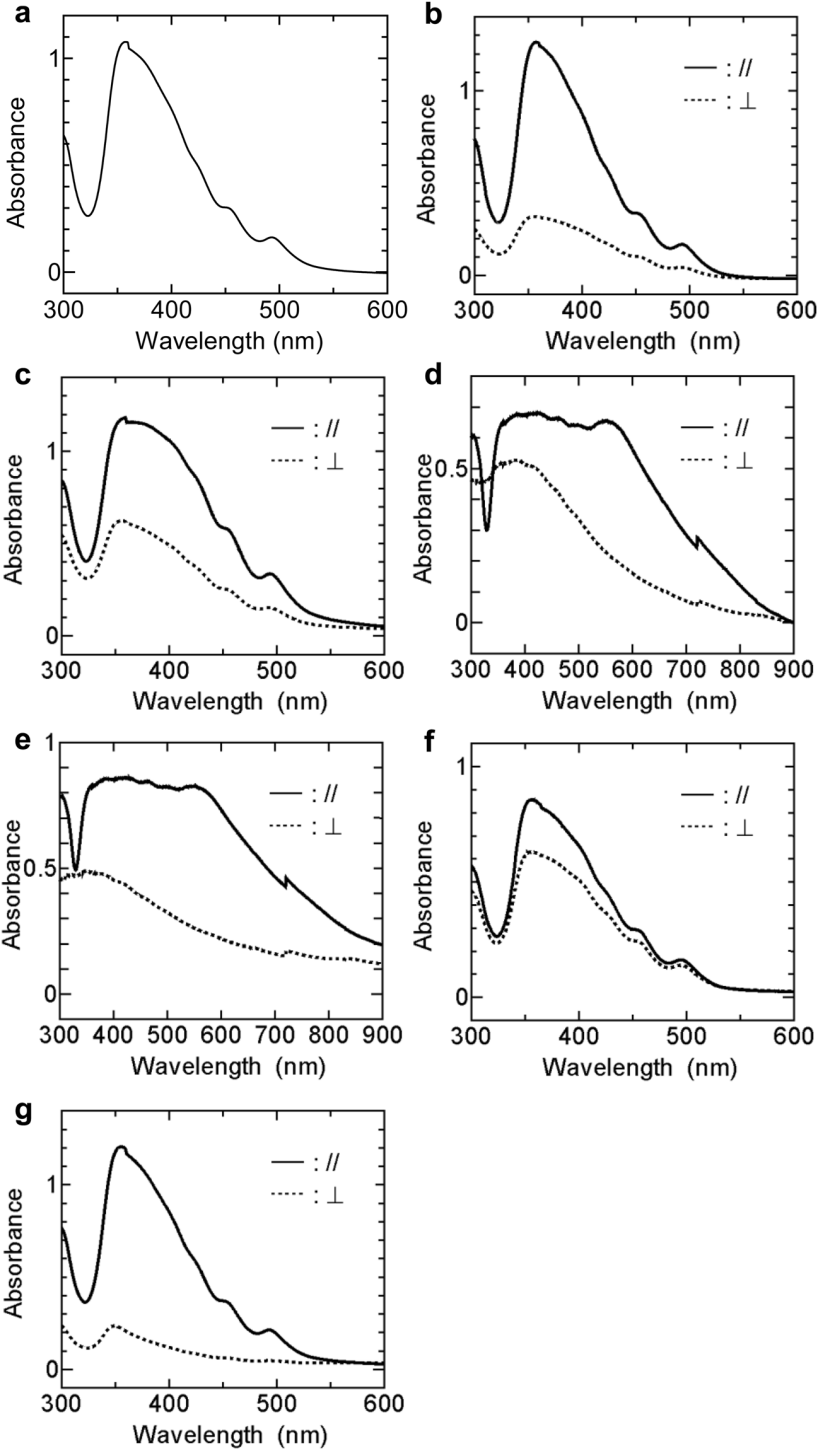
Polarised absorption spectra of the BP3T-OMe films. (**a**) Sample 1, (**b**) Sample 5, (**c**) Sample 6, (**d**) Sample 7, (**e**) Sample 8 (**f**) Sample 9 and (**g**) Sample 10. The marks “//” and “⊥” indicate the polarising directions that are parallel and perpendicular to the drawing direction, respectively. Note that the ranges of the horizontal axis are different in (**d**,**e**) and (**a**,**b**,**c**,**f**,**g**).

Figure 3 shows the polarised absorption spectra. The spectrum of Sample 1 (isotropic film) showed a maximum peak at ~360 nm [Fig. 3(a)]. In Samples 5 and 6 [Fig. 3(b,c)] the parallel components *A*_//_ of absorption spectrum relative to the drawing direction showed a maximum peak at ~360 nm and these maxima decreased in the perpendicular component *A*_⊥_.

For the postheat-treated films at 150 (Sample 7) and 200 °C (Sample 8) [Fig. 3(d,e)], the absorption bands at longer wavelength increased and the peak observed in Samples 5 and 6 around 360 nm became flat. These were related to the dark polarising micrograph images of the postheat-treated films [Fig. 2(e,f)].

From the polarised absorption spectra, we estimated the dichroic ratios *D* and the degrees of orientation *F* and summarise the results in Table 2. Of all the samples, Sample 10 [Fig. 3(g)] indicated the maximum *D* = 5.4 and *F* = 0.59 at 355 nm, indicating highly molecular orientation along the drawing direction.

**Table 2:** Dichroic ratios and degrees of orientations at the listed wavelengths.

Sample No.	Absorption		
	Wavelength (nm)	Dichroic ratio *D*	Degree of orientation *F*
5	357	4.0	0.50
6	359	1.9	0.23
7	359	1.3	0.08
8	359	1.7	0.19
9	356	1.4	0.11
10	355	5.4	0.59

### SEM observation

**Figure 4:**
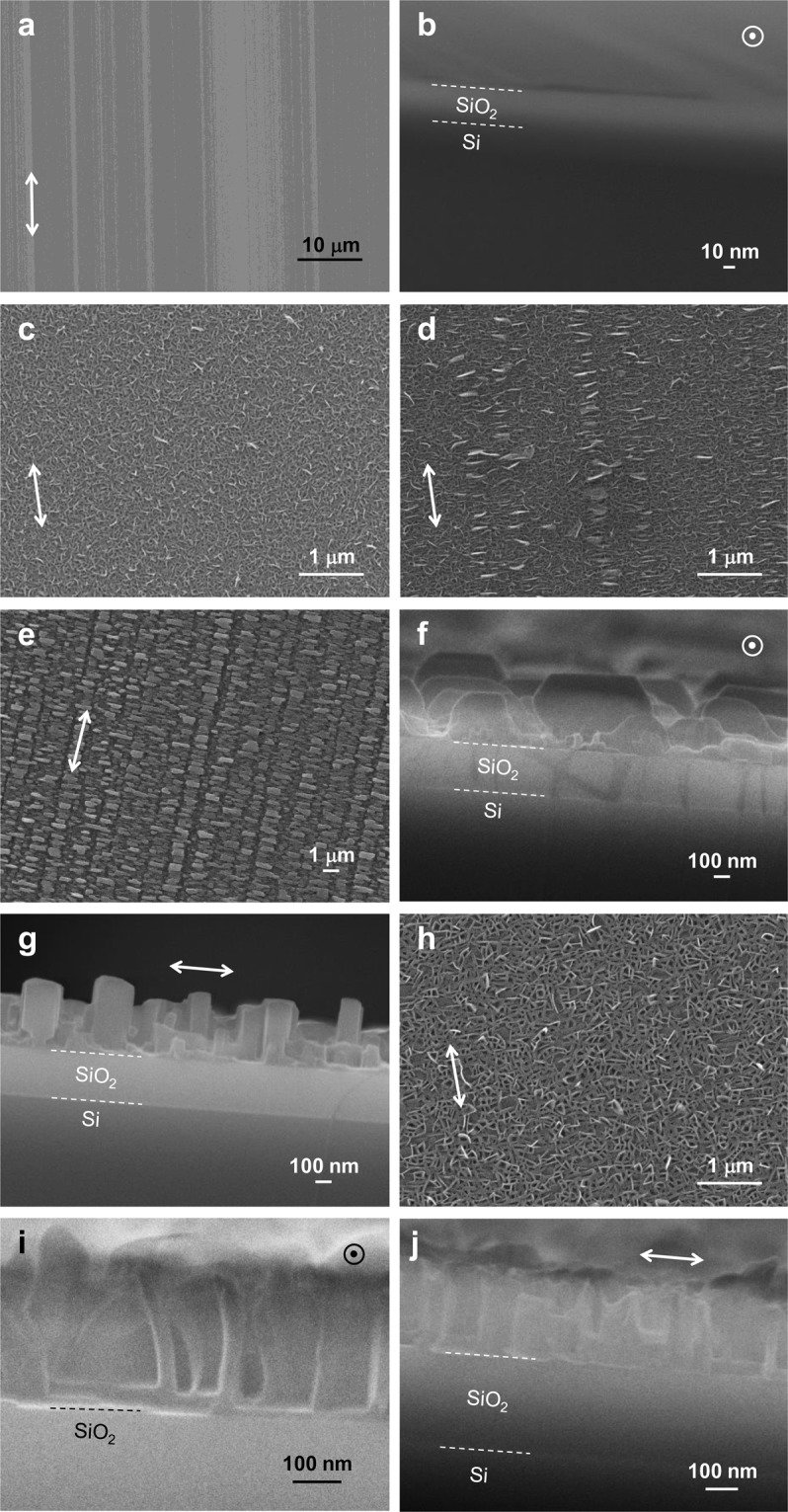
SEM images of the friction-transferred PTFE film and the BP3T-OMe films. (**a**) Top view and (**b**) cross-section of the friction-transferred PTFE film. Top views of (**c**) Sample 1, (**d**) Sample 5 and (**e**) Sample 8. (**f**,**g**) Cross-sections of Sample 8. (**h**) Top view of Sample 10. (**i**,**j**) Cross-sections of Sample 10. Double-headed arrows and circles with a dot in the middle indicate the drawing direction. The dotted lines indicate boundaries between Si and SiO_2_ or between SiO_2_ and the BP3T-OMe layer. Contrast and brightness of images (**b**,**f**,**g**,**i**,**j**) were adjusted to improve visualisation.

Figure 4 shows the SEM images of the friction-transferred PTFE film and the BP3T-OMe films deposited on the untreated substrate (Sample 1), the friction-transferred substrates without heat treatment (Sample 5), postheat-treated at 200 °C (Sample 8) and under substrate heating at 100 °C (Sample 10). Friction-transferred PTFE showed many straight lines on the substrate [Fig. 4(a)], as shown in the micrograph in our previous study^12^. The height of PTFE was ~10 nm [see Fig. 4(b)].

In Samples 1 and 5, BP3T-OMe films were composed of pleats [Fig. 4(c,d)]. Sample 5 indicated longitudinally-aligned pleats that were laterally stretched [Fig. 4(d)]. The friction-transferred PTFE lines were suggested to be under these aligned pleats because the alignment of the pleats was parallel to the drawing direction.

In Sample 8, the substrate surface was composed of longitudinally aligned lines made of horizontally long blocks [Fig. 4(e)]. Cross-section observation of Sample 8 indicated that these blocks were hexagonal cylinders standing on the substrate [Fig. 4(f)]. These hexagonal shapes mean the crystal growth through the postheat treatment^18,19^ and suggest that the crystallographic intermediate axis stood against the substrate surface, as indicated in the XRD measurements. From the point of view of molecular orientation, the molecules were most highly oriented in the film of Sample 8. Figure 4(g) indicates that these hexagonal cylinders were closely aligned along the drawing direction. This may affect the carrier mobility along this direction (vide infra).

Although larger pleats were observed in Sample 10 [Fig. 4(h)] than in Samples 1 and 5 [Fig. 4(c,d)], no aligned pleats were observed. The similar film morphologies composed of pleats in Samples 5 and 10 [Fig. 4(d,h)] may result in the similar XRD patterns [see Fig. S1(b,g), Supplementary Information] and the similar absorbance spectra [see Fig. 3(b,g)]. Figure 4(i,j) compare the cross-sections of Sample 10 cut along the perpendicular and parallel directions relative to the drawing direction, respectively. Although both the cross-section images show similar film morphology, the pleats seemed to spread along the drawing direction compared to the direction perpendicular to the drawing direction. This may also affect the carrier mobility as in the case with Sample 8. Another possible reason why Sample 10 showed the higher anisotropic optoelectrical properties (Tables 2, 3 and S2) despite no apparent aligned pleats is as follows: In the case of substrate heating, molecular alignment may improve at the location closer to the substrate rather than to the top surface along the film thickness direction. This is because the deposited molecules closer to the substrate were heated for a longer time.

**Table 3:** Mobilities in the linear and saturation regions, on/off ratio and subthreshold slope of devices.

Device No.	Mobility (cm^2^ V^−1^ s^−1^)		On/off ratio	Subthreshold slope (V dec^−1^)
	Linear region	Saturation region		
1	8.9 × 10^−3^	1.4 × 10^−2^	259	16.8
2	2.3 × 10^−3^	5.0 × 10^−3^	723	12.5
3	8.3 × 10^−3^	9.5 × 10^−3^	2010	9.1
4	2.7 × 10^−2^	2.6 × 10^−2^	2010	9.7
5-⊥	1.9 × 10^−4^	3.9 × 10^−4^	6	163.5
5-//	7.4 × 10^−3^	7.9 × 10^−3^	47	26.8
6-⊥	2.3 × 10^−4^	3.4 × 10^−4^	46	22.6
6-//	1.8 × 10^−3^	2.8 × 10^−3^	321	14.0
7-⊥	3.0 × 10^−4^	3.5 × 10^−4^	50	21.1
7-//	5.7 × 10^−3^	6.1 × 10^−3^	556	12.2
8-⊥	2.7 × 10^−4^	5.1 × 10^−4^	25	25.4
8-//	1.1 × 10^−2^	1.4 × 10^−2^	322	15.9
9-⊥	5.5 × 10^−4^	2.5 × 10^−4^	10	42.5
9-//	8.9 × 10^−3^	1.0 × 10^−2^	230	15.2
10-⊥	2.8 × 10^−4^	3.3 × 10^−4^	25	26.8
10-//	1.8 × 10^−2^	2.2 × 10^−2^	1926	10.7

### Molecular orientation

**Figure 5:**
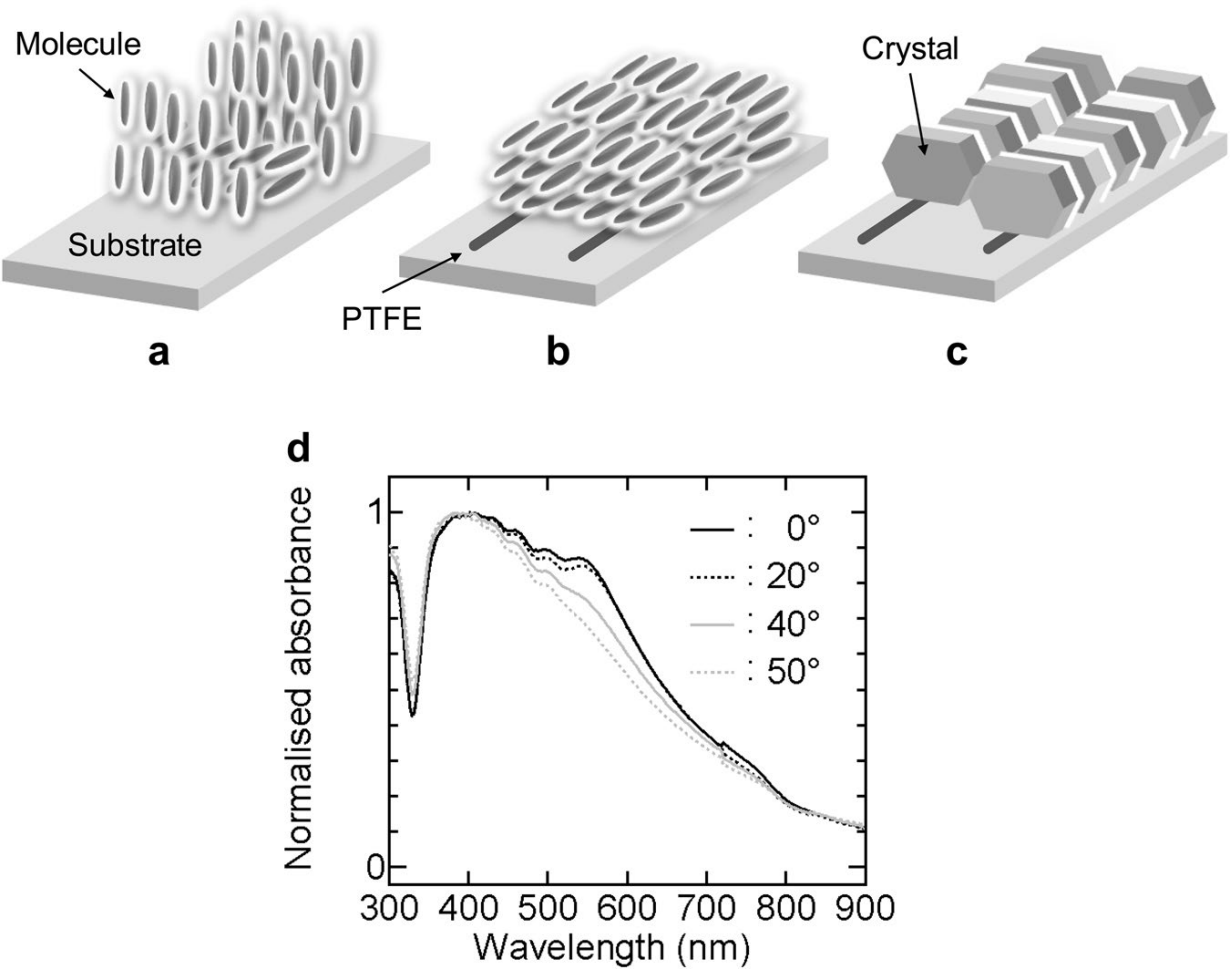
Schematic orientation of BP3T-OMe molecules deposited on (**a**) an untreated substrate and (**b**) a substrate with a friction-transferred PTFE layer. (**c**) Schematic alignment of BP3T-OMe crystals grown on a friction-transferred PTFE layer. (**d**) Incident-angle-dependent polarised absorption spectra of Sample 8. The angle 0° means that the irradiated light was perpendicular to the substrate surface.

From the results of polarising microscope observation, XRD and optical measurements and SEM observation, Fig. 5(a–c) schematically summarise molecular orientation of BP3T-OMe on the substrates. BP3T-OMe deposition on an untreated substrate (without friction transfer or heat treatment) produced clusters in which the molecules either stood or lay down against the substrate [Fig. 5(a)]. The lying molecules are predominant in clusters of the BP3T-OMe layer deposited on a friction-transferred substrate [Fig. 5(b)]. In the layer, the longer direction of the BP3T-OMe molecules is parallel to the drawing direction of PTFE. The aggregates of the clusters were supposed to form the pleats [see Fig. 4(d,h)].

Meanwhile, postheat treatment of the deposited BP3T-OMe layer on a friction-transferred substrate gave aligned hexagonal crystals along the transferred PTFE [Fig. 5(c)]. The longer direction of the molecules in the crystal is parallel to the drawing direction.

In TPCO crystals, the longer direction of molecules usually rises on the hexagonal surface, and the transition dipole moments are large along this direction^20^. The crystals absorbed most of light polarised parallel to the longer direction of molecules. The dark polarising micrographs [Fig. 2(e,f)] and the flat absorption spectra [Fig. 3(d,e)] of Samples 7 and 8 are attributed to the strong light absorption caused by the high degree of molecular orientation^21,22^. Figure 5(d) shows incident-angle-dependent polarised absorption spectra of Sample 8. The rotation axis was perpendicular to the three directions of light traveling, light polarisation and PTFE drawing. At 0°, the drawing direction was parallel to the light polarisation direction. With deviating the polarisation direction of light from 0° to 50°, the absorption spectra over 500 nm decreased, coming close to *A*_⊥_ [see Fig. 3(e)].

### OFET characteristics

The output characteristics are shown in Fig. S3 (Supplementary Information) for OFETs made from Samples 1–10. All OFETs indicated well-defined p-type conduction. We estimated carrier mobilities in the linear and saturation regions and summarise them in Table 3 and Fig. S5(a,b) (Supplementary Information). In the table, devices were indicated by sample number (integer) and the drawing direction relative to the channel length direction (parallel “//” or perpendicular “⊥”). For some devices with the PTFE layer friction-transferred along ⊥ direction, it is difficult to estimate the accurate mobility from the linear regions due to the disordered current increases around the origin. Hence, we used the mobility in the saturation regions for a fair comparison. The OFETs with postheat-treated films showed the following tendency. For the OFETs without friction-transferred PTFE layers (Devices 2–4), the mobility increased up to 2.6 × 10^−2^ cm^2^ V^−1^ s^−1^ in the saturation region (Device 4) with the postheat treatment temperature. However, the postheat treated sample at 100 °C (Device 2) showed the lower mobility than the unheated one (Device 1). The same was observed in the OFETs with the PTFE layer friction-transferred along // direction (Devices 5-//, 6-//, 7-// and 8-//). In contrast, the mobility was almost independent with the postheat treatment temperature for the OFETs with the PTFE friction-transferred along ⊥ direction (Devices 5-⊥, 6-⊥, 7-⊥ and 8-⊥). For substrate heating, the mobilities of both Devices 9-// (1.0 × 10^−2^ cm^2^ V^−1^ s^−1^) and 10-// (2.2 × 10^−2^ cm^2^ V^−1^ s^−1^) showed similar values to that of Device 8-// (1.4 × 10^−2^ cm^2^ V^−1^ s^−1^).

The mobility of Device 5-// (7.9 × 10^−3^ cm^2^ V^−1^ s^−1^ in the saturation region) was larger than that of Device 5-⊥ (3.9 × 10^−4^ cm^2^ V^−1^ s^−1^), as shown in Devices 6–10, but smaller than that of Device 1 (1.4 × 10^−2^ cm^2^ V^−1^ s^−1^). The mobility ratio of Device 5-// to Device 5-⊥ is ~20, indicating the high anisotropy. The increases in the drain current from the origin were observed in the devices having the channel length direction parallel to the drawing direction [see Fig. S3(f,h,j,l,n,p), Supplementary Information]. This indicates the good electrical contact between the Au electrodes and the BP3T-OMe film.

Among the devices, Devices 8-// and 10-// indicated the larger mobilities, and the latter device indicated the largest mobility of 2.2 × 10^−2^ cm^2^ V^−1^ s^−1^ in the saturation region. This value was almost 1.5 times as large as that of Device 1 (1.4 × 10^−2^ cm^2^ V^−1^ s^−1^) and ~67 times larger than that of Device 10-⊥ (3.3 × 10^−4^ cm^2^ V^−1^ s^−1^). In the case of Sample 8, the crystals were closely aligned along the drawing direction [Fig. 4(g)]. This makes the mobility along the drawing direction larger. This may cause ~27 times larger mobility of Device 8-// (1.4 × 10^−2^ cm^2^ V^−1^ s^−1^ in the saturation region) than that of Device 8-⊥ (5.1 × 10^−4^ cm^2^ V^−1^ s^−1^). Different from Samples 5 and 8, longitudinally aligned lines composed of laterally-stretched pleats or horizontally long blocks were not observed in Sample 10 [see Fig. 4(d,e,h)]. However, the largest polarisation ratio of the emissions [Fig. S2(f) and Table S2, Supplementary Information] and degree of orientation (Table 2) were also observed along with the largest mobility in Sample 10. This suggests that Sample 10 had the highly molecular orientation which was not directly observed by microscopic observation. As seen in Fig. 4(j), the continuously spread pleats along the drawing direction caused these results. Meanwhile, one possible reason that the mobilities were smaller along the direction perpendicular to the drawing direction than parallel to that direction is that the channel was interrupted by the discontinuous separation between adjacent alignments of pleats or hexagonal crystals. Device 8-// showed the slightly smaller mobility (1.4 × 10^−2^ cm^2^ V^−1^ s^−1^) than Device 10-// (2.2 × 10^−2^ cm^2^ V^−1^ s^−1^) in the saturation region, even though the molecules seemed to be highly aligned in Sample 8 than in Sample 10 (Tables 2 and S2). The slightly smaller mobility may be caused by chasms between the crystal blocks in Sample 8 [Fig. 4(e)]. These chasms might obstruct carrier transfer in the direction parallel to the channel. In contrast, the pleats seem to be in close contact with each other in Sample 10 [Fig. 4(h)].

We also evaluated the transfer characteristics of the devices in Fig. S4 (Supplementary Information) to examine the effect of the friction-transferred PTFE layer to interface characteristics of the channel layer. The OFETs with the friction-transferred PTFE layer showed anisotropy in on/off ratio and subthreshold slope [Table 3 and Fig. S5(c,d)]. Among Devices 1–8, the on/off ratio and subthreshold slope were improved in all postheat-treated OFETs (Devices 2–4 and 6–8) up to 2010 and 9.1 V dec^−1^ (Device 3), respectively. In samples with substrate heating (Devices 9 and 10), the higher temperature (Device 10-//) was more effective to improve both on/off ratio (1926) and subthreshold slope (10.7 V dec^−1^), which are comparable with the best results (Device 3).

These results indicate that, although insertion of a friction-transferred layer lowers the mobility, the heat treatments not only recover the mobility but also improve the on/off ratio and subthreshold slope. On the whole, the postheat-treated OFET at 200 °C (Device 8-//) and the OFET with substrate heating at 100 °C (Device 10-//) showed the better performance than that without a friction-transferred film or heat treatment (Device 1).

## Conclusion

In the present studies, we made highly-anisotropic microcrystalline array structures through thermal crystal growth of BP3T-OMe orientation films on friction-transferred PTFE layers. We heated substrates after BP3T-OMe deposition or deposited it on the heated substrates. The orientation film without heat treatment was also prepared. We examined optical properties and morphology of the resulting films. The BP3T-OMe molecules lying-down on the friction-transferred substrate were dominant and their molecular long axis was in agreement with the drawing direction. The non-heated films showed longitudinally-aligned pleats structure. On the other hand, the microcrystalline array structures which were highly-aligned along the drawing direction were observed when the substrate was heated at 200 °C after BP3T-OMe deposition (“postheat” treatment). The BP3T-OMe film deposited on the heated substrate at 100 °C (substrate heating) provided no aligned pleats structures but intriguingly indicated the highest degree of orientation.

Using these films, we fabricated OFETs. The OFETs having the channel length direction parallel to the drawing direction of PTFE showed a linear increase in the drain current from the origin, indicating the good electrical contact between the BP3T-OMe film and electrodes. The OFET with microcrystalline array films (non-heated films) indicated the mobility 1.4 × 10^−2^ cm^2^ V^−1^ s^−1^ (7.9 × 10^−3^ cm^2^ V^−1^ s^−1^) in the saturation region. This value was almost 27 times (20 times) larger than the corresponding value 5.1 × 10^−4^ cm^2^ V^−1^ s^−1^ (3.9 × 10^−4^ cm^2^ V^−1^ s^−1^) of the OFET having the channel length direction perpendicular to the drawing direction. Even though the films treated with substrate heating had no aligned pleats structures, its OFET indicated the highest mobility and anisotropy.

The results demonstrate that the heat treatments enhanced the crystallinity and carrier mobility of the BP3T-OMe orientation films on the friction-transferred PTFE substrates while retaining the high anisotropy, showing usefulness of the heat treatments for controlling crystallinity and orientation of TPCO molecules. We expect the microcrystalline array structures to be utilised in organic solar cells due to the high crystallinity, the strong light absorption property and the convex-concave surface patterns associated with an interpenetration structure.

## Methods

### Fabrication of friction-transferred PTFE layer

We used Si substrates (10 mm × 10 mm) covered with an SiO_2_ layer (300 nm in thickness) and quartz substrates (10 mm × 10 mm). We cleaned them sequentially in acetone, 2-propanol, ethanol and distilled water with an ultrasonic cleaner for 10 min each and dried them for ~30 min in an oven. Its temperature was set at 120 °C. We prepared a PTFE sheet (10 mm in width, 40 mm in length and 0.8 mm in thickness), folded it in half and made its crease flat by polishing with sandpaper. We pressed the half fold PTFE sheet with a compression pressure of 1.5 kgf cm^−2^ onto the substrate that was preliminarily heated at 150 °C, and mechanically rubbed the sheet once at a rate of 60 mm min^−1^ ^12,23^. The resulting friction-transferred area was ~8 mm × 8 mm. The micrograph and XRD pattern of the transferred PTFE layer were described in the literature^12^. The aligned PTFE polymer chains lay horizontally on the substrate^12^.

### Oriented TPCO films fabrication

We used an Ulvac vacuum coater VPC-260F and deposited 100-nm-thick BP3T-OMe films on the substrates with a deposition rate of 0.02 nm s^−1^ in the vacuum of ~10^−3^ Pa through a handmade mask (hole size: ~8 mm × 10 mm). We determined the thickness by using an Ulvac deposition controller CRTM-6000 with a quartz oscillator. For postheat treatment, we used a hot plate and controlled the temperature of its top panel. The postheat treatment was carried out in a nitrogen atmosphere to prevent material thermal oxidation. For substrate heating, we used a heating unit of VPC-260F.

We observed macroscopic morphology and orientation of the BP3T-OMe films with a Nikon Eclipse LV100 POL polarising microscope and a Rigaku RINT 2500 X-ray diffractometer, respectively. We measured diffractions (*θ*/2*θ*-scans) in the angle range 2*θ* = 2–60° using a Cu-Kα X-ray source (wavelength: 0.15418 nm). We observed microscopic film morphology using a JEOL JSM-7001F scanning electron microscope.

For the emission measurements, we used a Hamamatsu Photonics PMA-11 photonic multichannel analyser using the same setup as before^12,20^. We irradiated ultraviolet excitation light (330–380 nm) through an objective lens (magnification was 50 or 100) of Eclipse LV100 POL perpendicular to the BP3T-OMe film surface and measured emission perpendicular to that surface. We measured parallel and perpendicular components of the emissions to the drawing direction by placing a polariser between the BP3T-OMe film and PMA-11.

We measured the polarised light absorption of the BP3T-OMe films using a Shimadzu UV-3600 UV-VIS-NIR spectrophotometer. For this, we used the BP3T-OMe films deposited on the friction-transferred quartz substrates with various heat treatments (see Table 1). Two polarisers were located between the light source and the film and between the film and the detector and their polarising directions were set parallel to each other. We irradiated monochromatic light (wavelength range: 300–600 nm or 300–900 nm) perpendicular to the substrate and measured polarised absorption along the direction both parallel and perpendicular to the drawing direction. We estimated a dichroic ratio *D* = *A*_//_/*A*_⊥_ and a degree of orientation *F* = (*D* − 1)/(*D* + 2) from absorbance components *A*_//_ and *A*_⊥_ polarised along the directions parallel and perpendicular to the drawing direction, respectively. As an option, we measured incident-angle-dependent polarised absorption spectra for Sample 8. We manually rotated the sample on a handmade holder to vary an incident angle of the light from the normal (defined as 0°) to 50° in 10° step.

### OFET fabrication and *I*–*V* characteristics

We fabricated OFETs with the BP3T-OMe films deposited on the friction transferred SiO_2_/Si substrates with various heat treatments (see Table 1). We formed source and drain contacts (area: ~1 mm × 4 mm) by depositing 200-nm-thick Au layers with a deposition rate of 0.15 nm s^−1^ over a tungsten wire (50 μm in diameter) to make a channel of the OFET. The channel lengths and widths were ranged ~19–50 μm and ~0.8–1.1 mm, respectively.

We carried out *I*–*V* characteristics measurements using a Keithley 4200-SCS semiconductor characterisation system in a vacuum (~10^−3^ Pa) in the dark. We measured drain currents *I*_D_ with one of Au electrodes grounded as the source contact^24^ and applied direct current (DC) voltages *V*_DS_ ranging from 10 to −60 V (or 10 to −100 V) to the other Au electrode (used as the drain contact) with various DC gate voltages *V*_G_ from 10 to −60 V (or 10 to −100 V) as a parameter. We obtained the output characteristics from the *I*–*V* measurements, and then, transfer curves were constructed based on the output characteristics data. We estimated carrier mobilities *µ* in the linear and saturation regions by the following equations^25^:

1 $${I}_{{\rm{D}}}=\frac{W}{L}\mu {C}_{{\rm{i}}}({V}_{{\rm{G}}}-{V}_{{\rm{th}}}){V}_{{\rm{DS}}}$$

2 $${I}_{{\rm{D}}}=\frac{W}{2L}\mu {C}_{{\rm{i}}}{({V}_{{\rm{G}}}-{V}_{{\rm{th}}})}^{2}$$

where *W* is the channel width, *L* is the channel length, *C*_i_ is the insulator capacitance (per unit area), and the *V*_th_ is the threshold voltage. For the calculations, we neglected the effects of the PTFE layer because this layer was much thinner (~10 nm, vide supra) than SiO_2_ (300 nm).

We also calculated the on/off ratio and subthreshold slope of the devices from the transfer curves at *V*_DS_ = −60 V. The on/off ratio was calculated by dividing maximum *I*_D_ by minimum *I*_D_. The subthreshold slope was determined as an absolute value of the reciprocal value of a transfer curve slope in a subthreshold region, where the vertical axis is log(*I*_D_) and the horizontal axis is *V*_G_.

### Quantum chemical calculation

We estimated the molecular length of BP3T-OMe from the atomic geometry optimised by a molecular orbital (MO) calculation using the Gaussian 09 program^26^ with the B3LYP method and the 6–31 G(d) basis set. The molecular length was defined by the distance between the terminal hydrogens of the molecule [see Fig. 1(a)]. The length includes the van der Waals radius of a hydrogen atom (1.2 Å)^27^.

## Supplementary information

Supplementary Information
