# Supplementary material for: Microcrystalline array structures induced by heat treatment of friction-transferred organic semiconductor films
Source: Sci Rep. 2019 Jul 5;9:9739. doi: 10.1038/s41598-019-46212-w (PMC6611772; doi:10.1038/s41598-019-46212-w)
Supplement: Supplementary file 1 — Supplementary Information [file 41598_2019_46212_MOESM1_ESM.pdf]

Supplementary Information

Microcrystalline array structures induced by heat treatment of friction-transferred organic semiconductor films

Yuhi Inada*, Masashi Koda, Yuji Urabe, Toshifumi Katagiri, Takeshi Yamao*, Yuji Yoshida & Shu Hotta

S1. Synthesis of BP3T-OMe

General

Unless otherwise noted, reagents and solvents were purchased from commercial sources and used for experiments without further purification. 2,5-Bis(5-bromo-2-thienyl)thiophene (**4**) [1] and tetrakis(triphenylphosphine)palladium(0) [2] [$Pd(PPh_3)_4$] were synthesised according to literatures. BP3T-OMe and the intermediates were synthesized based on the synthetic method of 2,5-bis(4'-methoxybiphenyl-4-yl)thiophene (BP1T-OMe) [3]. IR spectra were taken on a JEOL JIR-6500 FT-IR spectrophotometer with finely pulverised samples dispersed and embedded in a potassium bromide matrix. Electron impact mass spectra were collected on a SHIMADZU GCMS-QP5000 mass spectrometer. Melting points were determined on a Seiko Instruments EXSTAR DSC 6220 thermal analysis system.

4-Bromo-4'-methoxybiphenyl (2)

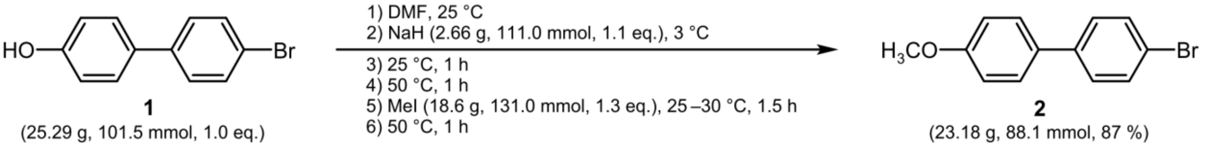

To a dry four-necked flask (500 mL) equipped with a mechanical stirrer were added 4'-bromobiphenyl-4-ol (**1**) (25.29 g, 101.5 mmol, 1.0 eq.) and dehydrated *N*,*N*-dimethylformamide (DMF; 250 mL). After the mixture was stirred at 25 °C until dissolved, the reaction vessel was cooled in an ice bath until the actual temperature of the mixture reached to 3 °C. After adding 4.44 g of 60wt% liquid paraffin dispersion of sodium hydride (NaH; 2.66 g, 111.0 mmol, 1.1 eq.) to the flask, the mixture temperature increased to 13 °C. After the mixture was stirred at 25 °C for 1 h, it was heated at 50 °C for 1 h to give a yellow homogeneous solution. The solution was cooled to 28 °C, and then, methyl iodide (MeI; 18.6 g, 131.0 mmol, 1.3 eq.) was slowly added over 5 min to increase the mixture temperature to 33 °C. After the mixture was stirred at 25–30 °C for 1.5 h, it was heated to 50 °C and kept for 1 h. After that, the reaction mixture was cooled to 25 °C and poured into a beaker of ice water (1 L) to yield a precipitate. The precipitate was collected by filtration and washed with water (200 mL). The wet powder was dissolved into a mixture of tetrahydrofuran (THF; 350 mL) and ethyl acetate (EtOAc; 210 mL), and then, dried over magnesium sulfate ($MgSO_4$; 90 g). $MgSO_4$ was removed by filtration and the solvent was evaporated under reduced pressure from the filtrate to provide a white solid (29.0 g). The solid was suspended to methanol (MeOH; 135 mL) and stirred for 1 h. After the filtration, the solid was washed with MeOH (50 mL) and dried at 40 °C under reduced pressure to afford the desired compound **2** (23.18 g, 88.1 mmol, 87%).

(4'-Methoxy-4-biphenyl)boronic acid (3)

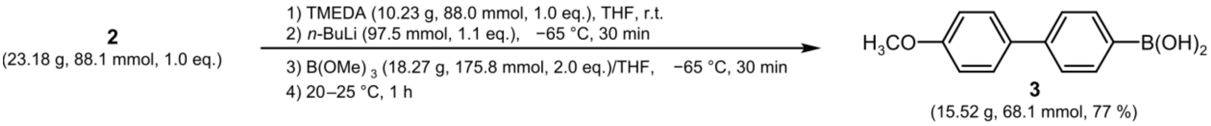

To a dry four-necked flask (1 L) equipped with a mechanical stirrer were added **2** (23.18 g, 88.1 mmol, 1.0 eq.) and dehydrated THF (680 mL). After **2** was dissolved at room temperature, *N*,*N*,*N'*,*N'*-tetramethylethylenediamine (TMEDA; 10.23 g, 88.0 mmol, 1.0 eq.) was added and the solution temperature was decreased to −65 °C. After that, *n*-butyllithium (*n*-BuLi; 2.6M hexane solution, 37.5 mL, 97.5 mmol, 1.1 eq.) was dropwise added over 15

min at −65 ± 2 °C and the solution was kept stirring for 30 min at the same temperature. To the mixture was added a trimethyl borate [$B(OMe)_3$; 18.27 g, 175.8 mmol, 2.0 eq.] in a dehydrated THF solution (55 mL) over 20 min at −65 ± 2 °C and the solution was kept stirring for 30 min at the same temperature. After the temperature of the reaction vessel was elevated to 20 °C over 30 min, the mixture was stirred at 20–25 °C for 1 h. The reaction mixture was cooled to 0 °C, and a 10wt% aqueous solution of sulfuric acid (H_2SO_4 aq.; 137 g) was dropwise added into the flask over 10 min, where the temperature was kept below 10 °C. After the mixture was stirred for 30 min, brine (340 mL) was added. Then, the mixture was poured into a separating funnel and the aqueous layer was separated. The aqueous layer was extracted with EtOAc, and the organic layers were combined and dried over $MgSO_4$. After filtration, the solvent was evaporated in vacuum until the solution weight became 163 g. Then, chloroform ($CHCl_3$; 340 mL) was added into the solution to give a precipitate. The precipitate was collected by filtration, washed with $CHCl_3$ (100 mL) and dried in *vacuo* at 40 °C to provide the target compound **3** (15.52 g, 68.1 mmol, 77%).

5,5''-Bis(4'-methoxybiphenyl-4-yl)-2,2':5',2''-terthiophene (BP3T-OMe) (5)

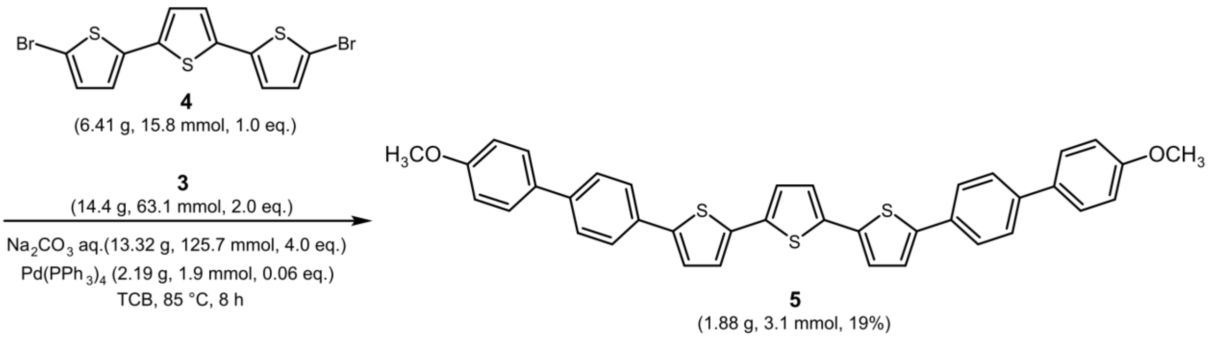

To a four-necked flask (2 L) equipped with a mechanical stirrer were added bronic acid **3** (14.4 g, 63.1 mmol, 2.0 eq.), **4** (6.41 g, 15.8 mmol, 1.0 eq.) and 1,2,4-trichlorobenzene (TCB; 1.5 L). After that, an aqueous solution of sodium carbonate (Na_2CO_3; 13.32 g, 125.7 mmol, 4.0 eq., 327 mL) was added. Then, $Pd(PPh_3)_4$ (2.19 g, 1.9 mmol, 0.06 eq.) was added and the mixture was reacted at 85 °C for 8 h. The reaction mixture was filtrated and the remaining solid was washed with water (200 mL). The thus-obtained solid was poured into acetone (200 mL), stirred for 1 h, washed with acetone (100 mL) on a filter and dried under reduced pressure to give crude (6.36 g). A part of the crude (2.93 g) was dissolved to TCB (1.7 L) at 200 °C, and then the solution was cooled and filtered. This purification process was also carried out for another part of the crude (2.93 g) and thus-obtained solids were combined, stirred in acetone (300 mL) for 30 min, washed with acetone (100 mL) on a filter and dried in vacuum to provide the target compound **5** (1.88 g, 3.1 mmol, 19%). IR (KBr): 3063, 1491, 1441, 1403, 1038, 824, 814, 805, 790 cm^{-1}; MS (EI): 612 [M]; mp: 401.0 °C.

References

1. Bauerle, P., Wurthner, F., Gotz, G. & Effenberger, F. Selective synthesis of α-substituted oligothiophenes. *Synthesis*, 1099–1103 (1993).
2. Coulson, D. R., Satek, L. C. & Grim, S. O. Tetrakis(Triphenylphosphine)Palladium(0). *Inorg. Synth.* **28**, 107–109 (1990).
3. Katagiri, T., Ota, S., Ohira, T., Yamao, T. & Hotta, S. Synthesis of thiophene/phenylene co-oligomers. V. Functionalization at molecular terminals toward optoelectronic device applications. *J. Heterocyclic Chem.*, **44**, 853–862 (2007).

S2. XRD measurements

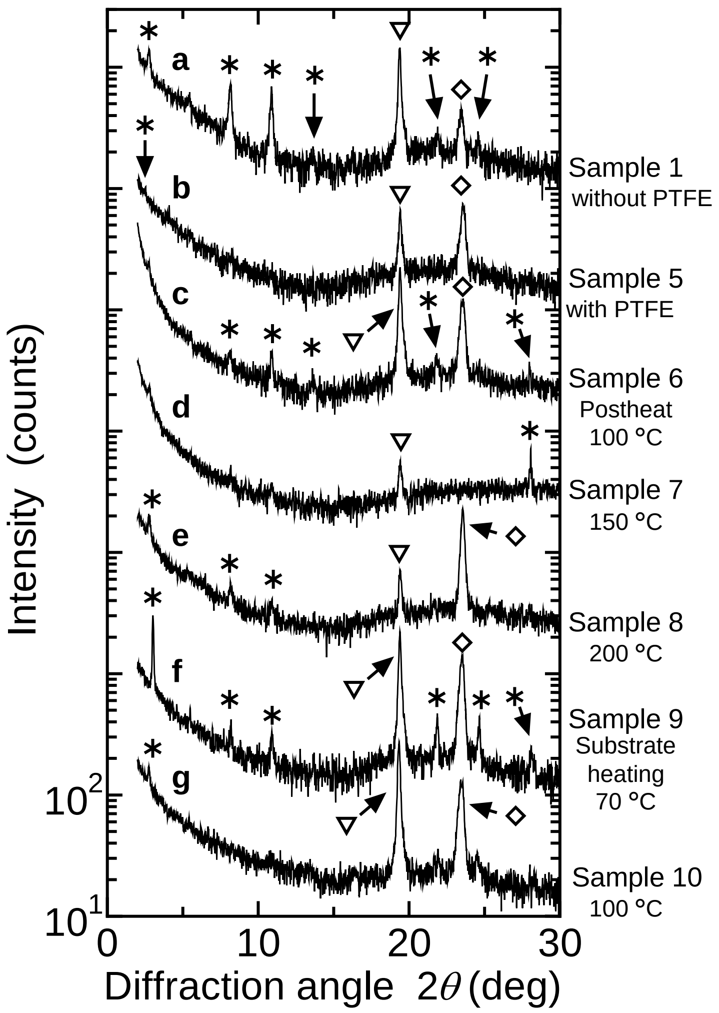

Figure S1. XRD patterns of the BP3T-OMe films. (**a**) Sample 1, (**b**) Sample 5, (**c**) Sample 6, (**d**) Sample 7, (**e**) Sample 8, (**f**) Sample 9 and (**g**) Sample 10. The peaks marked with asterisks, triangles and diamonds correspond to the diffractions associated with separations of ~3.2, ~0.46 and ~0.38 nm, respectively (see Table S1). The raw intensity data were smoothed using a three-point adjacent average. The diagrams are shifted in the longitudinal direction to avoid overlapping.

Table S1. Major peak positions 2θ for the XRD patterns of the BP3T-OMe films. The table includes the diffraction orders n and plane separations d.

Sample No.	2θ (degree)	n	d (nm)
1	2.79	1	3.167
	8.17	3	3.247
	10.88	4	3.253
	13.65	5	3.244
	19.39	1	0.458
	21.87	8	3.251
	23.46	1	0.379
	24.58	9	3.259
5	2.77	1	3.189
	19.40	1	0.458
	23.60	1	0.377
6	8.09	3	3.279
	10.90	4	3.247
	13.59	5	3.258
	19.39	1	0.458
	21.80	8	3.261
	23.59	1	0.377
	27.98	10	3.189
7	19.40	1	0.458
	28.05	10	3.181
8	2.77	1	3.189
	8.18	3	3.243
	10.90	4	3.247
	19.40	1	0.458
	23.54	1	0.378
9	3.02	1	2.925
	8.18	3	3.243
	10.91	4	3.244
	19.39	1	0.458
	21.87	8	3.251
	23.52	1	0.378
	24.67	9	3.248
	28.08	10	3.178
10	2.75	1	3.213
	19.33	1	0.459
	23.43	1	0.380

S3. Polarised emission measurements

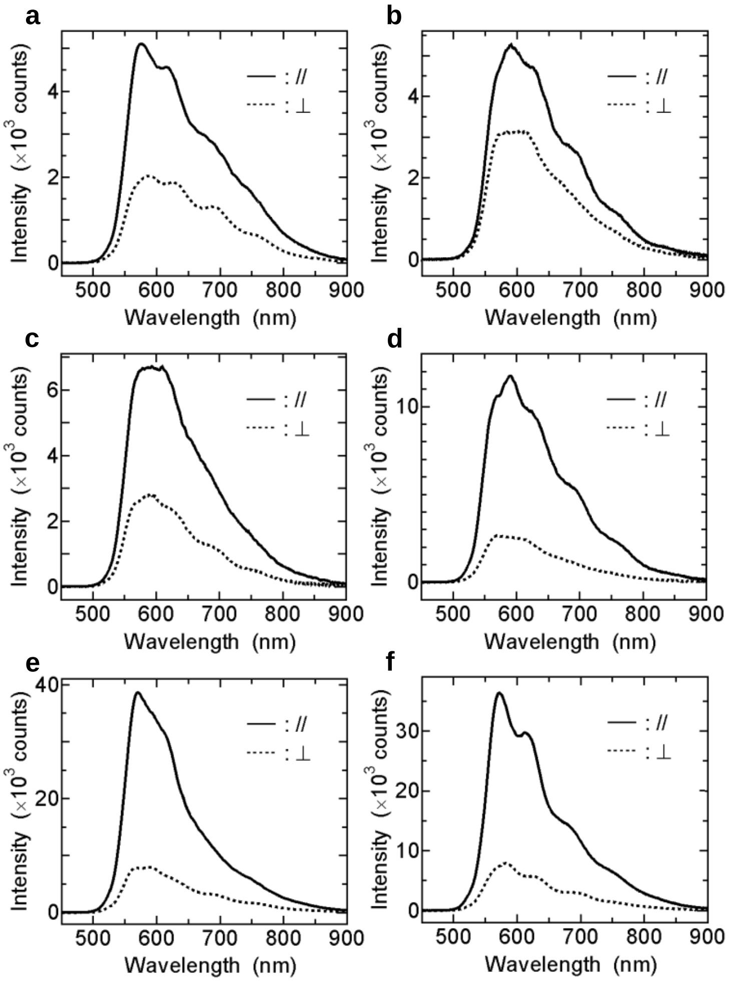

Figure S2. Polarised emission spectra of the BP3T-OMe films. (**a**) Sample 5, (**b**) Sample 6, (**c**) Sample 7, (**d**) Sample 8, (**e**) Sample 9 and (**f**) Sample 10. The marks "//" and "⊥" indicate the polarising directions that are parallel and perpendicular to the drawing direction, respectively.

Table S2. Polarisation ratios of polarised emission spectra. These were calculated as the ratios of the parallel emission component to the perpendicular one relative to the drawing direction at the listed wavelengths.

Sample No.	Emission	
	Wavelength (nm)	Polarisation ratio
5	573	2.7
	615	2.5
6	590	1.7
7	592	2.4
8	591	4.5
9	572	5.0
10	571	4.9
	614	5.2

S4. OFET characteristics

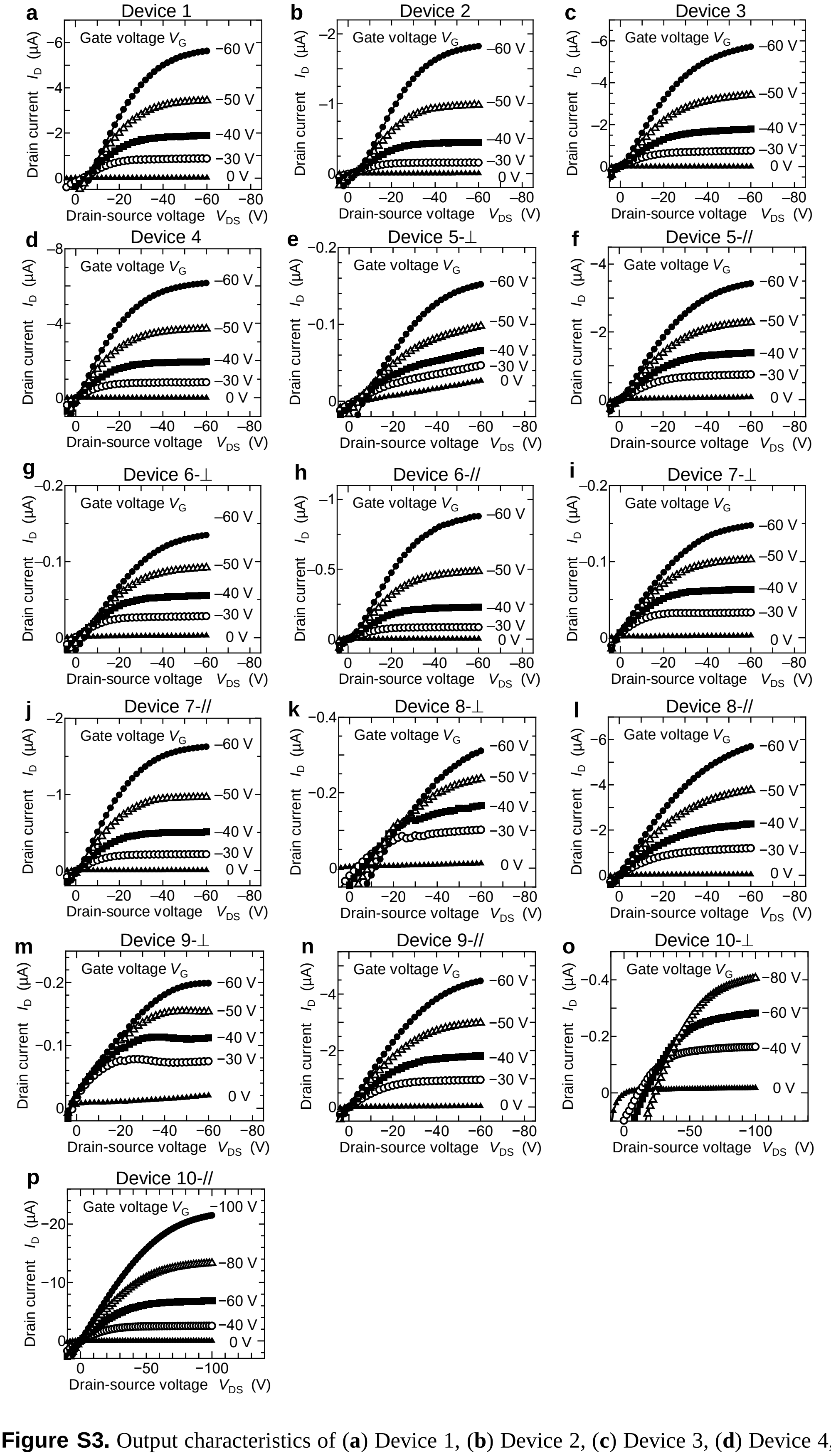

Figure S3. Output characteristics of (**a**) Device 1, (**b**) Device 2, (**c**) Device 3, (**d**) Device 4, (**e**) Device 5-⊥, (**f**) Device 5-//, (**g**) Device 6-⊥, (**h**) Device 6-//, (**i**) Device 7-⊥, (**j**) Device 7-//, (**k**) Device 8-⊥, (**l**) Device 8-//, (**m**) Device 9-⊥, (**n**) Device 9-//, (**o**) Device 10-⊥ and (**p**) Device 10-//.

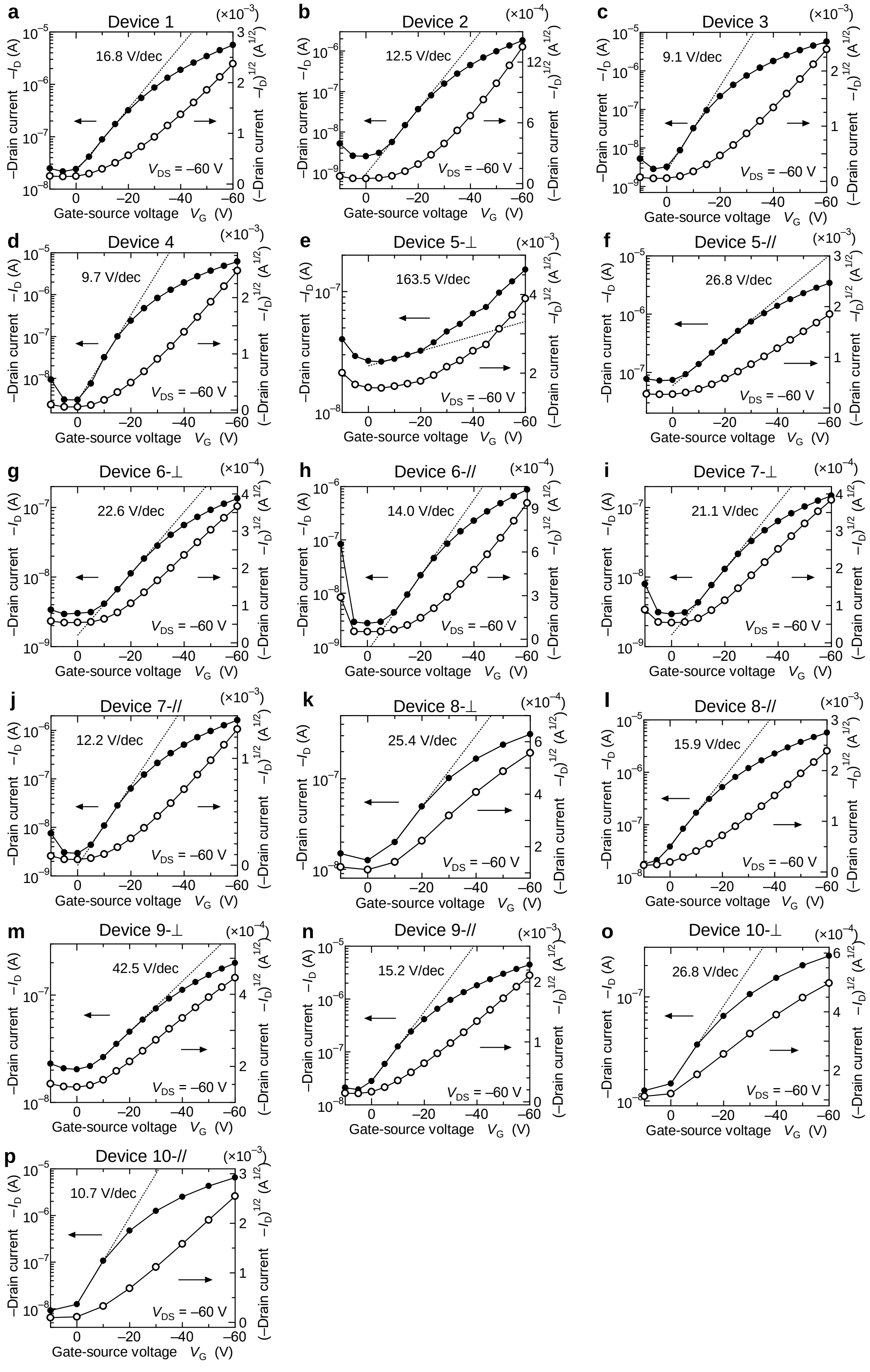

Figure S4. Transfer characteristics of (**a**) Device 1, (**b**) Device 2, (**c**) Device 3, (**d**) Device 4, (**e**) Device 5-⊥, (**f**) Device 5-//, (**g**) Device 6-⊥, (**h**) Device 6-//, (**i**) Device 7-⊥, (**j**) Device 7-//, (**k**) Device 8-⊥, (**l**) Device 8-//, (**m**) Device 9-⊥, (**n**) Device 9-//, (**o**) Device 10-⊥ and (**p**) Device 10-// at $V_{DS} = -60$ V.

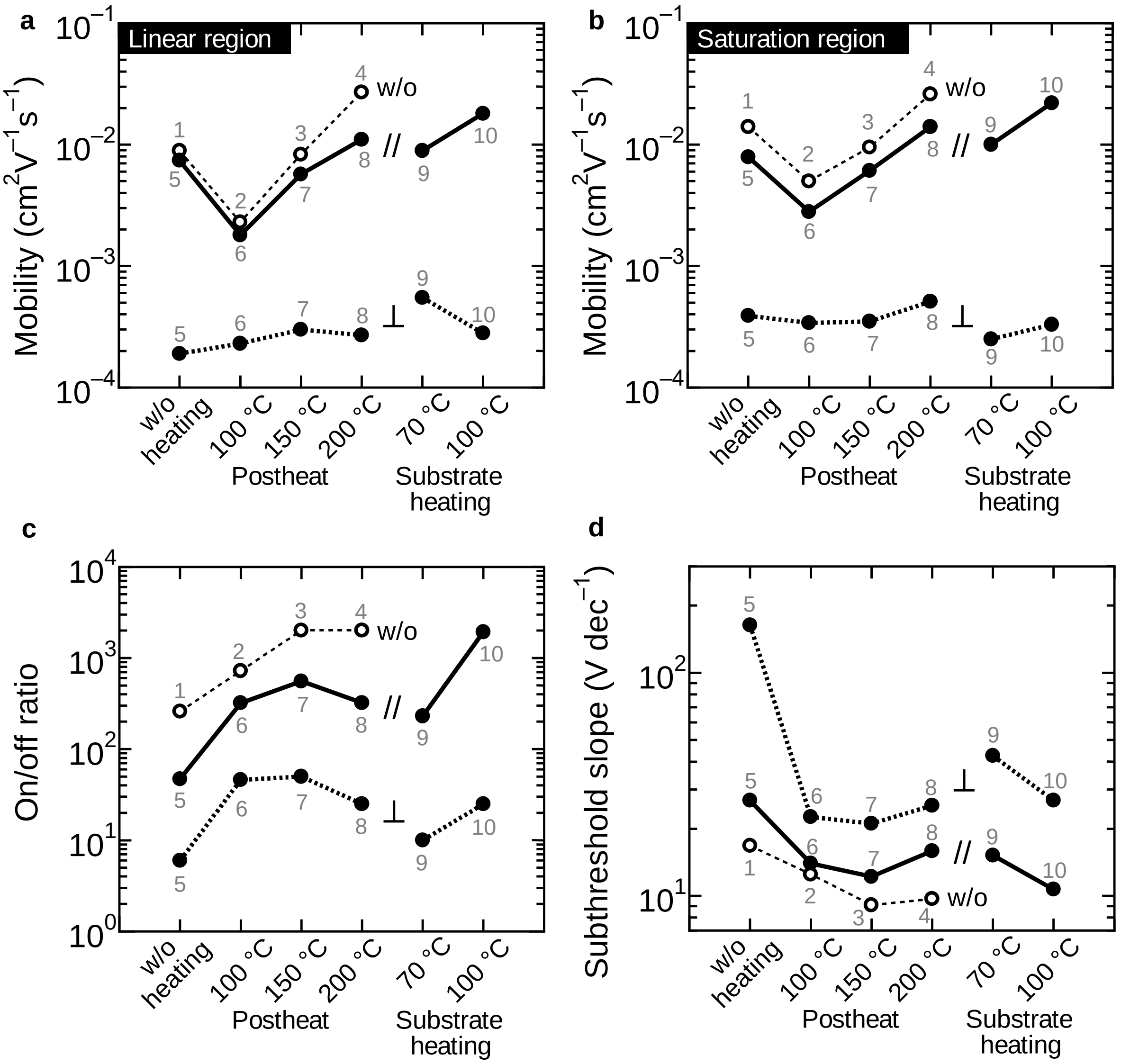

Figure S5. Comparisons of mobility in the (**a**) linear and (**b**) saturation regions, (**c**) on/off ratio and (**d**) subthreshold slope. The grey number (integer) in the figures denotes the device No.
